# Supplementary material for: Does Insomnia Increase the Risk of Suicide in Hospitalized Patients with Major Depressive Disorder? A Nationwide Inpatient Analysis from 2006 to 2015
Source: Behav Sci (Basel). 2022 Apr 19;12(5):117. doi: 10.3390/bs12050117 (PMC9137701; doi:10.3390/bs12050117)
Supplement: Supplementary file 1 [file behavsci-12-00117-s001.zip › behavsci-1430502-supplementary.pdf]

Table S1. ICD-9 code for major depressive disorders (primary diagnosis) before and after propensity score matching.

-	Original Cohort		Propensity Matched Cohort	
**ICD-9 Code for Major Depressive Disorder**	**MDD without Insomnia (N = 2924594)**	**MDD with Insomnia (N = 139061)**	**MDD without Insomnia (N = 276496)**	**MDD with Insomnia (N = 139061)**
29620	17.90%	16.30%	16.40%	16.30%
29621	0.10%	0.20%	0.20%	0.20%
29622	1.60%	1.60%	1.60%	1.60%
29623	6.60%	6.80%	6.80%	6.80%
29624	5.20%	4.30%	4.30%	4.30%
29625	0.10%	0.10%	0.10%	0.10%
29626	0.00%	0.00%	0.00%	0.00%
29630	14.70%	12.80%	12.80%	12.80%
29631	0.40%	0.30%	0.40%	0.30%
29632	6.20%	5.90%	5.90%	5.90%
29633	34.20%	39.40%	39.20%	39.40%
29634	12.70%	12.10%	12.10%	12.10%
29635	0.30%	0.40%	0.40%	0.40%
29636	0.00%	0.00%	0.00%	0.00%

Table S2. ICD-9 code for insomnia.

ICD-9 Code
04672
32700
32701
32702
32709
78051
78052
V695
